# Masculine Discrepancy Stress, Emotion-Regulation Difficulties, and Intimate Partner Violence

**DOI:** 10.1177/0886260516650967

**Published:** 2016-05-24

**Authors:** Danielle S. Berke, Dennis E. Reidy, Brittany Gentile, Amos Zeichner

**Affiliations:** 1 University of Georgia, Athens, GA, USA; 2 Centers for Disease Control and Prevention, Atlanta, GA, USA

**Keywords:** masculine discrepancy stress, intimate partner violence, emotion regulation, multiple mediation

## Abstract

Research suggests that masculine socialization processes contribute to the perpetration of intimate partner violence (IPV) by men. Although this research has traditionally focused on men who strongly adhere to traditional gender norms, men who negatively evaluate themselves as falling short of these norms (a construct termed *masculine discrepancy stress*) have proven to be at increased risk of IPV perpetration. Likewise, men experiencing problems with emotion regulation, a multidimensional construct reflecting difficulties in effectively experiencing and responding to emotional states, are also at risk of IPV perpetration. In the present research, we tested the hypothesis that the link between discrepancy stress and IPV perpetration is mediated via difficulties in emotion regulation. Three hundred fifty-seven men completed online surveys assessing their experience of discrepancy stress, emotion-regulation difficulties, and history of IPV perpetration. Results indicated that discrepancy-stressed men's use of physical IPV was fully mediated by emotion-regulation difficulties. In addition, emotion-regulation difficulties partially mediated the association between discrepancy stress and sexual IPV. Findings are discussed in terms of the potential utility of emotion-focused interventions for modifying men's experience and expression of discrepancy stress and reducing perpetration of IPV.

Intimate partner violence (IPV) constitutes a significant and preventable public health problem with consequences ranging from loss of productivity, negative physical and mental health outcomes, and even death. Indeed, in a recent large-scale epidemiological study, IPV resulted in an estimated 2,340 deaths per year—accounting for 14% of all homicides (U.S. Department of Justice, Bureau of Justice Statistics, 2011). Importantly, seventy percent of the victims in this survey were women. It is necessary to note that men are also affected by partner violence. Moreover, evidence suggests that men and women may experience similar motivators and precipitators of violence perpetration (Langhinrichsen-Rohling, 2010). However, national survey data indicate women are physically victimized by male intimate partners at higher rates and experience increased negative consequences. For example, nearly 13.4% of women versus 3.5% of men have been injured physically as a consequence of IPV (Breiding et al., 2014). These data suggest the importance of gender socialization and masculine-relevant processes for understanding and preventing violence against women.

## Masculine Discrepancy Stress and Intimate Partner Violence

Both cognitive and experiential factors of gender socialization have been posited to play a role in men's aggression. From a social learning perspective, cognitive beliefs and attitudes permissive of violence are modeled to boys and men by adults and other social figures (Lefkowitz, Eron, Walder, & Huesmann, 2013). Thus, IPV can be understood as one context in which men attempt to express socialized masculine beliefs and norms (Anderson, 2005; Connell, 2005). Alternatively, men's aggression may be understood in terms of experiential factors such as stress associated with violations of masculine norms (Franchina, Eisler, & Moore, 2001; Jakupcak Lisak, & Roemer, 2002). Indeed, rather than being passive recipients of dominant masculine norms and ideology, a growing body of literature suggests that boys and men vary in terms of the degree to which they conform to gendered expectations (Berke, Sloan, Parrot, & Zeichner, 2011; Chu, 2014; Mahalik et al., 2003; Reigeluth & Addis, 2016). Although nonconformity to masculine norms has been associated with aspects of adaptive psychological functioning (Chu, 2014; Mahalik et al., 2003), men who fail to conform to masculine gender roles may also be at increased risk of engagement in IPV (Vandello & Bosson, 2013). This risk can be understood in terms of Pleck's theoretical formulation of *discrepancy stress* (Pleck, 1981, 1995). Pleck proposed that violations of gender standards may be associated with negative consequences for men's self-esteem and psychological well-being, given that such violations often result in negative social feedback and internalized negative self-judgments. Moreover, Pleck proposed that the experience of masculine discrepancy stress (DS) may lead individuals to overconform to masculine norms including expressions of aggression and dominance. In a recent study, Reidy and colleagues provided support for this proposal by identifying DS as a risk factor for heterosexual men's perpetration of IPV against a current female partner, independent of other masculinity-related variables (Reidy, Berke, Gentile, & Zeichner, 2014). This study was the first to offer explicit empirical support for a relationship between DS and IPV. Although this study provided important confirmation of Pleck's theory, the mechanisms driving this relationship have yet to be fully explicated. As such, the current study aims to build on these preliminary findings by expanding understanding of potential processes by which DS confers risk of IPV perpetration.

Two contemporary empirical frameworks provide a basis from which to generate hypotheses regarding mediators of the association between DS and violence: *precarious manhood* (Vandello, Bosson, Cohen, Burnaford, & Weaver, 2008) and *fear-based learning* (e.g., Addis, 2011; Kimmel, 2010). In their laboratory studies on precarious manhood, Vandello and colleagues (2008) have garnered significant support for the notion that masculine status is easily revoked or lost. First, given that physical aggression is often an effective demonstration of manhood (Vandello & Bosson, 2013), engaging in IPV may provide an opportunity for men who experience DS to temporarily regain masculine status. Second, IPV perpetration may emerge from a process of fear-based learning given that violations of masculine norms are often punished with physical threat or social condemnation (Franchina et al., 2001; Zeman & Garber, 1996). Fear and threat of negative social consequences may motivate men to modify their behaviors to align more closely with (aggressive) norms, thereby allowing them to avoid future punishment (e.g., Addis, 2011; Kimmel, 2010). Considering these frameworks in synthesis, aggression may also emerge as a dysregulated expression of affective arousal produced by the experience of DS. As such, Jakupcak and colleagues have suggested that IPV perpetration may function as a means of regulating the distressing emotions stemming from pressures to conform to precarious masculine norms (Jakupcak et al., 2002), a proposition aligned with laboratory research indicating that aggression may in fact confer a mood-regulating function (Bushman, Baumeister, & Phillips, 2001).

## Emotion-Regulation Difficulties and Intimate Partner Violence

However, aggressive behavior may be associated with a general pattern of emotion *dys*regulation, and the potential affect regulating effects of aggression may be limited to the short term. For example, Gardner and Moore (2008) proposed that aggression acts akin to avoidance in the context of clinical anger, permitting the individual to inhibit the direct expression or acknowledgment of negative affect. At the same time, attempts to inhibit or suppress emotion have the ironic effect of increasing autonomic arousal (e.g., Gross & Levenson, 1993, 1997; Ohira et al., 2006) and negative affect (Dalgleish, Yiend, Schweizer, & Dunn, 2009; Gross & John, 2003). Each of these factors has been shown to significantly diminish an individual's ability to resolve difficult interpersonal situations (Ben-Zur, 2009), thereby increasing the likelihood of interpersonal violence (Roberton, Daffern, & Bucks, 2012). Thus, the ability to adaptively regulate emotional states is a key intervention target for reducing IPV perpetration. Indeed, research suggests that failures in dispositional self-control (i.e., the degree to which individuals are able to control impulses across time and situation) are an important predictor of IPV perpetration. For example, in a series of studies, Finkel and colleagues demonstrated that participants high in dispositional self-control were less likely to perpetrate IPV. Moreover, they showed that participants who completed a brief training regime designed to bolster self-regulatory resources exhibited less violent inclinations (Finkel, DeWall, Slotter, Oaten, & Foshee, 2009). These data suggest that interventions addressing emotion-regulation difficulties have the potential to effectively reduce IPV perpetration.

Given evidence for the multidimensional nature of emotion regulation, specification of the precise domain(s) of emotion-regulation difficulties that underlie men's IPV perpetration is essential to the development of effective, tailored interventions. Contemporary models of emotion regulation include acceptance, understanding, and awareness of emotions as well as the ability to inhibit impulsive behaviors and flexibly modulate emotions in the service of superordinate goals (e.g., Gratz & Roemer, 2004). Difficulties in several of these domains have been shown to predict aggression in general and IPV perpetration, in particular. For example, Gratz and Roemer (2004) found significant positive correlations between men's self-reported frequency of IPV perpetration and perceived limited access to emotion-regulation strategies. They also found IPV perpetration to significantly correlate with difficulties inhibiting impulsive behaviors when distressed and engaging in goal-directed behaviors in the context of negative affect. In addition, limited emotion awareness and low distress tolerance have been shown to mediate the link between negative affect and aggression in men (Donahue, Goranson, McClure, & Van Male, 2014).

## Masculinity and Emotion-Regulation Difficulties

Emotion-regulation difficulties have been identified as a prominent component operating in both men and women's perpetration of IPV (Langhinrichsen-Rohling, 2010); however, these difficulties are most certainly expressed in gender-specific ways. Indeed, contemporary theoretical and empirical work indicates that emotions themselves are an emergent product of socialization processes brought to bear on core affect. Core affect can be conceptualized as “a neurophysiological barometer of the individual's relation to an environment at a given point in time” (Barrett, 2006, p. 31). Thus, specific experiences and expressions of emotion are thought to emerge as a function of norms and assumptions an individual brings to bear on his or her physiological reaction to a particular context. That is, the interpretations that a man makes about the experience of negative affect (such as the negative affect concomitant with DS) are likely influenced by gender norms. Prevailing gender norms governing emotional expression hold that men should suppress their emotions, with the exception of anger (Chaplin, Cole, & Zahn-Waxler, 2005; Eisler & Skidmore, 1987; Grossman & Wood, 1993). Indeed, recent research indicates that boys and men are often punished for displaying vulnerable emotions such as sadness, guilt, and fear (O'Neil, 2008). As such, emotional avoidance and suppression may serve as a strategy for nonconforming men to reassert alignment with precarious masculine status and to manage fear and threat of social condemnation (Krugman, 1995; Tull, Jakupcak, Paulson, & Gratz, 2007).

The pressure to over-control emotional experiences may be particularly salient for men experiencing DS, who are likely prone to gender-threatening thoughts. These thoughts, in turn, may produce “vulnerable” emotions (e.g., sadness, anxiety), which would exacerbate DS given the incongruence between these emotions and masculine norms. Consequently, discrepancy-stressed men may be more likely to avoid or suppress these vulnerable emotions to express emotions more congruent with masculine norms (i.e., anger), despite the arousing effects of emotional suppression (Gross & Levenson, 1993, 1997; Ohira et al., 2006). Therefore, discrepancy-stressed men are likely susceptible to the very difficulties in emotion regulation underlying risk of IPV perpetration.

## Current Study

Evidence suggests that men who negatively evaluate themselves as falling short of masculine norms may be at increased risk of IPV perpetration (Reidy et al., 2014). Importantly, theoretical evidence suggests that this risk may be conferred via difficulties in emotion regulation that underlie DS (Krugman, 1995; Tull et al., 2007). However, this proposition has yet to be examined empirically. Nor have specific domains of emotion-regulation difficulties related to discrepancy stress-linked IPV been explored. Thus, the purpose of the current study was to build on theoretical and empirical literature regarding the experience of DS, its impact on emotion regulation, and attendant risk of IPV perpetration. Moreover, we sought to elucidate specific etiological pathways by which DS may give rise to physical and sexual aggression in intimate relationships. The multivariate assessment of emotion-regulation difficulties in the context of discrepancy-stressed men's IPV provides a unique opportunity to identify (and eventually target) modifiable mechanisms of perpetration.

**Figure 1:**
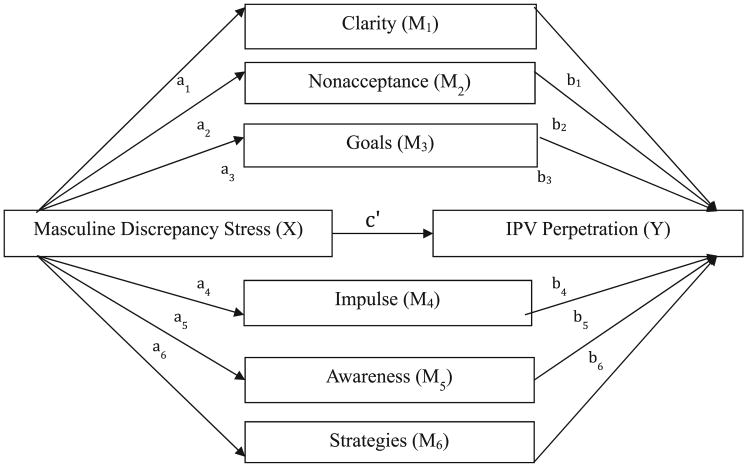
Path diagram of multiple mediator model. *Note.* Intimate Partner Violence stands for Physical Assault/Sexual Coercion score on the Conflict Tactics Scale–2; a_1_-a_6_ and b_1_-b_6_ refer to regression coefficients reported in Tables 1 to 3. M1-M6 are subscales of the Difficulties in Emotions Regulation Scale.

The current study constitutes a secondary analysis of survey data collected by Reidy and colleagues in 2012 with the aim of testing the hypothesis that emotion-regulation difficulties mediate the relationship between DS and IPV. Baseline evidence for a significant relationship between DS and IPV by men was first established in this sample and has been reported elsewhere (Reidy et al., 2014). In the present investigation, we sought to expand these findings by explicating the role of emotion-regulation difficulties in this relationship. As such, the following hypotheses were specified: Given evidence that hypomasculine men may be prone to avoid or over-control their emotional experiences in the context of DS, we hypothesized a significant relationship between masculine DS and difficulties in emotion regulation. Furthermore, consistent with extant literature (Donahue et al., 2014; Gratz & Roemer, 2004), we predicted that emotion-regulation difficulties would be associated with IPV. Last, we predicted that emotion-regulation difficulties would mediate the relationship between DS and IPV. See Figure 1 for a graphic depiction of these hypotheses.

## Method

### Participants and Procedure

Participants were 405 U.S. men recruited via Amazon's Mechanical Turk (MTurk) website who indicated they “had been in an intimate relationship within the prior 12 months.” MTurk permits the collection of national data from individuals via an online method that proffers valid and reliable data with more diversity in samples than traditional convenience samples (Paolacci & Chandler, 2014). Individuals were compensated US$2.00 each for completion of the questionnaires. The university institutional review board (IRB) approved all consent statements, materials, and procedures used in this study. Portions of this data have been reported elsewhere (Reidy et al., 2014; Reidy, Brookmeyer, Gentile, Berke, & Zeichner, 2016).

### Measures

#### Demographics questionnaire

Participants responded to a series of questions about age, ethnicity, marital status, relationship history, self-identified sexual orientation, and level of education.

#### Masculine Discrepancy Stress Scale

Respondents answered 5-point scale questions (1 = “strongly agree” to 7 = “strongly disagree”) pertaining to the experience of DS (i.e., “I wish I was more manly,” “I wish I was interested in things that other guys find interesting,” “I worry that people judge me because I'm not like the typical man,” “Sometimes I worry about my masculinity,” “I worry that women find me less attractive because I'm not as macho as other guys”). This scale (Reidy et al., 2016) has been shown to predict risky sexual behavior in men and injurious assaults against non-intimate victims (Reidy, Berke, Gentile, & Zeichner, 2015; Reidy et al., 2016) and demonstrated good internal consistency in the current sample (α = .86).

#### Conflict Tactics Scale–2 (CTS-2)

The CTS-2 (Straus, Hamby, Boney-McCoy, & Sugarman, 1996) instructs respondents to rate the frequency with which they engaged in aggressive relationship behavior as described on the form (between “never” and “more than 20 times”) and provides an option for “not in my current or most recent relationship, but it has happened before.” We used the full Physical Assault and Sexual Coercion subscales of the CTS-2 to assess men's perpetration of IPV in their current or most recent relationship. Sample items from the Physical Assault subscale include “Slapped my partner” and “Kicked my partner.” The Sexual Coercion subscale includes such items as “Insisted on sex when my partner did not want to (but did not use physical force)” and “Used force (like hitting, holding down, or using a weapon) to make my partner have sex.” Previous psychometric evidence supports the internal reliability and validity of the CTS-2 as a measure of relationship aggression (Straus et al., 1996). Cronbach's alphas for Physical Assault and Sexual Coercion sub-scales in the present sample were .97 and .83, respectively.

#### Difficulties in Emotion-Regulation Scale (DERS

The DERS (Gratz & Roemer, 2004) is a 36-item self-report measure comprised of six subscales that assess various dimensions of emotion-regulation difficulties. Participants indicate how often each item applies to them on a 5-point scale, with 1 as “almost never” (0%-10%) and 5 as “almost always” (91%-100%). Higher scores indicate greater difficulties in emotion regulation. Regarding its subscales, items on the *Clarity* subscale reflect the extent to which individuals know the emotions they are experiencing (e.g., “I am confused about how I feel”). Items on the *Nonacceptance* subscale assess lack of acceptance of emotions (e.g., “When I'm upset, I become embarrassed for feeling that way”). The *Goals* subscale reflects an inability to engage in goal-directed behavior when distressed (e.g., “When I'm upset, I have difficulty getting things done”), while the *Impulse* subscale reflects impulse control difficulties when experiencing negative affect (e.g., “When I'm upset, I feel out of control”). An *Awareness* subscale contains items measuring difficulties attending to ongoing emotional experiences (e.g., “I pay attention to how I feel”). Last, the *Strategies* subscale assesses limited access to strategies for effective regulation of emotions (e.g., “When I'm upset, I believe that there is nothing I can do to make myself feel better”). The DERS has demonstrated adequate convergent validity with established measures of emotion-regulation difficulties and emotional avoidance and has been demonstrated to be predictive of IPV in an undergraduate sample (Gratz & Roemer, 2004). Cronbach's alphas across subscales in the present sample ranged from .80 (Clarity) to .93 (Nonacceptance).

## Results

### Data Reduction and Sample Demographic Characteristics

Given our goal of investigating heterosexual men's violence toward female partners, 41 men who did not identify as exclusively heterosexual (i.e., gay, queer, bisexual, or transgender) on the demographics questionnaire were excluded from analyses. In addition, we removed seven respondents who were more than three standard deviations from the mean time of completion on the survey (*M* = 34.0, *SD* = 38.3, range = 5.3-677.8). The final analytic sample comprised 357 men with a mean age of 28.1 (*SD* = 6.9). The sample was largely consistent with the general U.S. population in terms of ethnicity (73% Caucasian, 11% Asian, 8% Black or African American, and 8% Hispanic/Latino) and income (median = US$37,500; mode = US$55,000; range = ≦ US$5,000 to ≥US$100,000), but slightly more educated (median = some college; mode = some college; range = ≦7 years of school to graduate school or professional training) compared with the general population of men (U.S. Census Bureau, 2010-2012). Regarding relationship history, 61% of the sample had never been married, 33.4% endorsed one marriage, 4% endorsed two marriages, and 1.6% endorsed three or more marriages. Finally, 11.2% of the sample reported a history of mental health diagnosis.

### Association Between Masculine Discrepancy Stress and Difficulties in Emotion Regulation

Path analyses of the association between DS and difficulties in emotion regulation revealed moderate positive associations for all DERS subscales. In other words, higher levels of DS were predictive of greater difficulty in all dimensions of emotion regulation. The results of these analyses are detailed in Table 1.

### Association Between Difficulties in Emotion Regulation and Intimate Partner Violence

To ascertain the unique influence of each domain of the DERS on men's engagement in IPV, two simultaneous regression models were constructed in which we regressed each IPV outcome (i.e., Physical Assault and Sexual Coercion) on all six DERS subscales. Results are provided in Table 2. For Physical Assault, unique effects emerged for the Clarity, Goals, and Impulse subscales. Men with difficulties clarifying the emotions they are experiencing (Clarity) and difficulties remaining in control of one's behavior when distressed (Impulse), reported significantly greater use of physical assault against their female intimate partners, regardless of relative difficulties or strengths in other emotion-regulation domains. In contrast, men with difficulties engaging in goal-directed behavior when distressed (Goals) reported less use of physical assault. Similarly, for Sexual Coercion, the Impulse subscale emerged as a positive predictor of violence while the Goals subscale was a negative predictor.

### Mediation Analyses

Path analyses specifying Physical Assault and Sexual Coercion as outcome variables were tested in separate multiple mediator models to estimate indirect effects between DS and IPV perpetration via difficulties in emotion-regulation domains. All variables were standardized prior to analyses to permit meaningful comparison across coefficients. Bootstrap estimates were calculated to examine indirect effects, as this method does not require assumption of normality in the sampling distribution (Preacher & Hayes, 2008). All indirect pathways were calculated and tested using bootstrap estimates based on 10,000 resamples (Hayes, 2013). As shown in Table 3, bootstrap estimates revealed significant indirect effects of DS on Physical Assault via difficulties in emotion regulation considered in total, 95% confidence interval = [.05, .16]. Moreover, examination of specific indirect effects suggests that this total effect was driven by the indirect effect of DS on IPV perpetration via Clarity [.01, .11], Goals [−.07, −.01], Impulse [.03, .11], and Strategies, [.00, .07]. Bootstrap estimates of the indirect effect of discrepancy stress on Sexual Coercion also produced significant coefficients for the DERS total scores [.04, .16], along with the Clarity [.00, .11], Goals [−.05, −.01], and Impulse [.03, .13] subscales.

Bootstrap estimates of the direct effect coefficients of DS on IPV perpetration controlling for all DERS subscales were also calculated (see Table 2). For Physical Assault, the direct effect (c′) did not reach significance, [−.04, .19]. In the context of the significant indirect effects reported above, these data support full mediation. When considering Sexual Coercion as the outcome variable, a direct effect for DS remained [.01, .24], even when controlling for all DERS subscales.

## Discussion

The purpose of the current study was to build on extant literature regarding risk factors for men's perpetration of violence against their intimate partner (Reidy et al., 2014). Specifically, we aimed to integrate gender socialization theories of IPV with a widely accepted and clinically relevant model of emotion-regulation difficulties by focusing on the consequences of masculine discrepancy stress (DS). This goal was pursued in the service of identifying modifiable risk and protective factors underlying discrepancy-stressed men's perpetration of IPV. It was expected that emotion-regulation difficulties would mediate the relationship between men's experience of DS and perpetration of IPV. Results of the current study revealed an interesting pattern of findings, highlighting the salience of emotion-regulation difficulties for discrepancy-stressed men's risk of violence perpetration.

First, we found a significant relationship between DS and emotion-regulation difficulties. Extant theory and data suggest that the anxiety-producing effects of perceiving oneself as falling short of precarious masculine standards render men vulnerable to emotion-regulation difficulties by encouraging the overreliance on avoidant emotion-regulation skills and impeding the development of more flexible skills for experiencing and expressing emotions directly (e.g., Cohn, Jakupcak, Seibert, Hildebrandt, & Zeichner, 2010; Tull et al., 2007; Wong, Pituch, & Rochlen, 2006). However, this study is the first to link DS in particular to emotion-regulation difficulties. Pertinently, DS was a significant predictor of all measured dimensions of emotion-regulation difficulties, indicating that distress specific to the belief that one is falling short of prevailing standards of masculinity may be a particularly potent impediment to effective emotion regulation.

These findings may be associated with the norm violating nature of DS itself. In other words, men who are distressed by self-perceived deficiencies in their masculinity must contend with vulnerable emotions. These emotions may, in turn, be perceived as further violations of masculinity given prevailing norms dictating toughness and restricted emotional expression (e.g., Eisler & Skidmore, 1987; Grossman & Wood, 1993). The experience of DS may, thus, motivate men to hyper-conform to masculine norms (Pleck, 1981, 1995) in terms of denying, avoiding, or otherwise suppressing emotion, which may have an unintended consequence of dysregulating less intentional emotional processes including the ability to control impulses and engage in goal-directed behavior when distressed. Overtime, this pattern may limit the ability of discrepancy-stressed men to identify their emotions with clarity and to develop a repertoire of skills for reducing distress, resulting in an overreliance on aggression as a (maladaptive) emotion-regulation strategy.

Second, consistent with previous studies and emotion-regulation models of aggression, results of the current study revealed significant associations between emotion-regulation difficulties and IPV perpetration (Gratz & Roemer, 2004; Pond et al., 2012). Importantly, given the analytic design of the current study, our results provide novel data regarding the *unique* effect of each domain of emotion-regulation difficulties on IPV. Specifically, we found that the Clarity and Impulse subscales significantly predicted the frequency at which men in our sample utilized physical IPV, even when controlling for their competencies in other emotion-regulation domains. These results suggest that men who struggle to accurately interpret and label emotional experiences and those with problems inhibiting impulsive behavior when distressed may be at particular risk of physical IPV perpetration. Difficulties inhibiting impulsive behavior when distressed also predicted the frequency of sexual IPV perpetration. These results suggest that difficulties in any one of those significant emotion-regulation domains may be sufficient, in isolation, to engender risk of IPV.

Indeed, results of the current study were consistent with full mediation for physical assault, such that the relationship between DS and physical IPV was fully explained by discrepancy-stressed men's emotion regulation difficulties. However, not all emotion-regulation domains contributed equally to this effect. Our findings indicate that a specific subset of emotion-regulation difficulties drove discrepancy-stressed men's risk of engagement in physical IPV against their intimate partner. Notably, in the context of DS, difficulty identifying one's emotional experience with clarity (Clarity), inhibiting automatic responses when distressed (Impulse), and believing that few strategies for coping with strong negative emotion are available (Strategies) appear to underlie risk of physical IPV perpetration. The emergence of significant indirect effects of discrepancy stress on physical IPV perpetration via these specific difficulties in emotion-regulation integrates, extends, and clarifies theory regarding the etiology of men's physical IPV. In contrast to physical IPV, discrepancy stress accounted for the men's sexual IPV above and beyond the effects of emotion regulation. Although, sexual IPV was partially explained by men's difficulties with emotional clarity and impulsivity, results of the present study indicate that discrepancy-stressed men may continue to be at risk of perpetration of sexual IPV even in the absence of emotion-regulation difficulties.

The finding that emotion-regulation difficulties fully mediated the relationship between DS and physical IPV but not sexual IPV is an important and novel contribution of the present study. This finding provides preliminary evidence that physical and sexual IPV may differ in important ways, particularly as they relate to gender-socialization processes. Specifically, these findings suggest that sexual IPV is more directly related to the performance of masculinity than physical IPV and therefore, less affected by an individual's emotion-regulation skills. Abundant evidence supports a relationship between hypermasculinity and sexually aggressive behavior (e.g., Abbey & McAuslan, 2004; Murnen, Wright, & Kaluzny 2002; Swartout, 2013). As such, sexual IPV may be a particularly effective strategy for enacting masculine norms of power, dominance, and virility, irrespective of the perpetrator's emotion-regulation skills. Thus, men who experience distress in response to perceiving themselves to be hypomasculine may use sexual IPV as a strategy to (re) construct their masculinity.

Notably, regarding the indirect effect of DS on physical and sexual IPV perpetration via difficulties engaging in goal-directed behavior when distressed (Goals), mediation results were in the opposite direction of other significant emotion-regulation difficulties. In other words, discrepancy-stressed men were more likely to perpetrate IPV when goal-oriented. Because aggression is seen as a highly salient method of demonstrating manhood (Vandello & Bosson, 2013), this pattern may indicate that violence is congruent with the goals of discrepancy-stressed men, particularly if that goal is to reassert masculine status.

### Limitations

Several limitations of this study warrant comment. First, given the cross-sectional design of this study, causal determinations regarding interrelations among assessed variables may not be made. More research is needed to fully evaluate the theoretical proposition that masculine socialization processes in general, and DS in particular, temporally precede emotion-regulation difficulties and downstream IPV. For example, although this study provided compelling theoretical and statistical support for the supposition that engaging in IPV may provide an opportunity for men who experience DS to temporarily regain masculine status, it is also possible that engaging in IPV could, in fact, function to exacerbate DS for men who endorse proscriptive norms about violence toward women (i.e., “never hit a woman”). Moreover, the present results do not address the developmental trajectory by which gender-based learning and associated stress inform one another. Although there is evidence that boys learn to perform gender “in the context of social learning that not only shapes adherence to gender norms, but also produces anxiety, fear, and other forms of restricted social and emotional functioning” (see Reigeluth & Addis, 2016, p. 1), future research investigating boys and adolescents in addition to men (especially studies with longitudinal designs) is needed to elucidate the temporal contingencies by which masculine socialization informs emotion expression and downstream aggression. Such methodology could help clarify whether DS precipitates emotion-regulation difficulties or whether men with emotion-dysregulation difficulties are more likely to experience DS.

Second, utilization of self-report measures may skew the actual prevalence of IPV perpetration. Given social proscriptions against violence toward women (Basow, Cahill, Phelan, Longshore, & McGillicuddy-DeLisi, 2007; Taylor & Sorenson, 2005), men in the current sample may have underreported the frequency of violence perpetration due to socially desirable responding. Given the dependence of this study on self-report of relationship status, it is also not possible to assure the veracity of participant report of IPV as having occurred in the context of an intimate relationship within the past year.

Last, participants in this sample were a relatively homogeneous sample of men. Although MTurk data typically proffers greater diversity than undergraduate convenience samples, the generalizability of future research would be increased by the inclusion of greater diversity across ethnicity, race, and class, particularly as masculinity is likely constructed in intersection with these variables. The mean participant age of this sample, 28.1 (*SD* = 6.9), is younger than that of studies utilizing clinical samples of men court-mandated to treatment for use of IPV. As such, associations among DS, emotion-regulation difficulties, and IPV should be tested in a clinical sample of older adults, to determine whether similar patterns emerge among participants with more extensive histories of violence and behavioral health difficulties. Such investigations are necessary to ascertain whether IPV interventions focused on reducing DS, and associated emotion dysregulation may be effective in clinically relevant contexts.

### Clinical Implications and Conclusion

Despite these limitations, the current study has significant implications for the development of targeted violence-prevention interventions. As emotion regulation may be conceptualized as a modifiable constellation of skills (Linehan, 1993), the elucidation of specific emotion-regulation difficulties as potential mechanisms underlying masculinity-linked engagement in IPV is of particular utility. Specifically, results of the current study suggest that for men who are distressed by self-perceptions of falling short of masculine norms, interventions focusing on building skillfulness in clear emotion identification and the development of a broad and flexibly applied armamentarium of emotion-regulation strategies may be particularly salient avenues for limiting IPV in general and physical assault in particular. The literature linking masculine aggression to contemporary emotion-regulation models remains in a nascent stage, warranting much more empirical and translational study. Nonetheless, the current research has critical implications for the importance of facilitating men's access to opportunities for learning more balanced and flexible expressions of emotions and masculine identity as a means of reducing risk of engagement in IPV.

## Figures and Tables

**Table 1:** Regression Effects of DS on DERS Subscales.

Regression Results	Coeff	*t*	*p*
(a_1_) Clarity regressed on DS	**.33**	**6.16**	**.00**
(a_2_) Nonacceptance regressed on DS	**.22**	**3.98**	**.00**
(a_3_) Goals regressed on DS	**.17**	**2.97**	**.00**
(a_4_) Impulse regressed on DS	**.21**	**3.82**	**.00**
(a_5_) Awareness regressed on DS	**.14**	**2.57**	**.01**
(a_6_) Strategies regressed on DS	**.29**	**5.40**	**.00**

*Note.* Letters in parentheses refer to regression paths in Figure 1. Significant coefficients are bolded. DS = masculine discrepancy stress; Coeff = completely standardized path coefficient; DERS = Difficulties in Emotion-Regulation Scale.

**Table 2:** Regression of Unique Effects of Each DERS Subscale on IPV Perpetration, Total Effects of DS on IPV, and Direct Effects of DS on IPV, Controlling for DERS.

Outcome Variable	Physical Assault			Sexual Coercion		
		
Regression Results	Coeff	*t*	*p*	Coeff	*t*	*p*
(b_1_) IPV regressed on Clarity	**.17**	**2.28**	**.02**	.15	1.90	.06
(b_2_) IPV regressed on Nonacceptance	−.07	−1.00	.32	−.06	−0.83	.41
(b_3_) IPV regressed on Goals	**−.21**	**−3.31**	**.00**	**−.17**	**−2.60**	**.01**
(b_4_) IPV regressed on Impulse	**.31**	**3.97**	**.00**	**.32**	**3.96**	**.00**
(b_5_) IPV regressed on Awareness	.01	0.10	.92	.07	1.11	.27
(b_6_) IPV regressed on Strategies	.12	1.24	.22	.04	0.43	.67
(c) IPV regressed on DS	**.17**	**3.13**	**.00**	**.23**	**3.92**	**.00**
(c′) IPV regressed on DS controlling for all DERS subscales	.07	1.32	.19	**.13**	**2.18**	**.03**

*Note.* Letters in parentheses refer to regression paths in Figure 1. Significant coefficients are bolded. DERS = Difficulties in Emotion-Regulation Scale; IPV = intimate partner violence as measured by the Conflict Tactics Scale–2; DS = masculine discrepancy stress; Coeff = completely standardized path coefficient.

**Table 3:** Results of Path Analyses Estimating the Indirect Effects of DS on IPV via Difficulties in Emotion Regulation.

Outcome Variable	Physical Assault			Sexual Coercion		
		
Indirect Effects	Coeff	*SE*	95% CI	Coeff	*SE*	95% CI
Total indirect effects						
(a_1_b_1_ + a_2_b_2_ + a_3_b_3_ + a_4_b_4_ + a_5_b_5_ + a_6_b_6_)	**.10**	**.03**	**[.05, .16]**	**.09**	**.03**	**[.04, .16]**
via Clarity (a_1_b_1_)	**.06**	**.02**	**[.01, .11]**	**.05**	**.03**	**[.00, .11]**
via Nonacceptance (a_2_b_2_)	−.02	.01	[−.04, .00]	−.02	.02	[−.06, .01]
via Goals (a_3_b_3_)	**−.03**	**.01**	**[−.07, −.01]**	**−.03**	**.01**	**[−.05, −.01]**
via Impulse (a_4_b_4_)	**.06**	**.02**	**[.03, .11]**	**.07**	**.02**	**[.03, .13]**
via Awareness (a_5_b_5_)	.00	.01	[−.02, .02]	.01	.01	[−.01, .03]
via Strategies (a_6_b_6_)	**.03**	**.02**	**[.00, .07]**	.01	.02	[−.03, .05]

*Note.* Letters in parentheses refer to regression paths in Figure 1. Significant coefficients are bolded. DS = masculine discrepancy stress; IPV = intimate partner violence as measured by the Conflict Tactics Scale–2; Coeff = completely standardized path coefficient; CI = confidence interval.
